# Quantitative Evaluation of Temporal Regularizers in Compressed Sensing Dynamic Contrast Enhanced MRI of the Breast

**DOI:** 10.1155/2017/7835749

**Published:** 2017-08-28

**Authors:** Dong Wang, Lori R. Arlinghaus, Thomas E. Yankeelov, Xiaoping Yang, David S. Smith

**Affiliations:** ^1^School of Science, Nanjing University of Science and Technology, Nanjing, Jiangsu, China; ^2^Department of Mathematics, Vanderbilt University, Nashville, TN, USA; ^3^Vanderbilt University Institute of Imaging Science, Vanderbilt University Medical Center, Nashville, TN, USA; ^4^Institute for Computational and Engineering Sciences and Departments of Biomedical Engineering and Internal Medicine, The University of Texas at Austin, Austin, TX, USA; ^5^Department of Mathematics, Nanjing University, Nanjing, Jiangsu, China

## Abstract

**Purpose:**

Dynamic contrast enhanced magnetic resonance imaging (DCE-MRI) is used in cancer imaging to probe tumor vascular properties. Compressed sensing (CS) theory makes it possible to recover MR images from randomly undersampled *k*-space data using nonlinear recovery schemes. The purpose of this paper is to quantitatively evaluate common temporal sparsity-promoting regularizers for CS DCE-MRI of the breast.

**Methods:**

We considered five ubiquitous temporal regularizers on 4.5x retrospectively undersampled Cartesian in vivo breast DCE-MRI data: Fourier transform (FT), Haar wavelet transform (WT), total variation (TV), second-order total generalized variation (TGV_*α*_^2^), and nuclear norm (NN). We measured the signal-to-error ratio (SER) of the reconstructed images, the error in tumor mean, and concordance correlation coefficients (CCCs) of the derived pharmacokinetic parameters *K*^trans^ (volume transfer constant) and *v*_*e*_ (extravascular-extracellular volume fraction) across a population of random sampling schemes.

**Results:**

NN produced the lowest image error (SER: 29.1), while TV/TGV_*α*_^2^ produced the most accurate *K*^trans^ (CCC: 0.974/0.974) and *v*_*e*_ (CCC: 0.916/0.917). WT produced the highest image error (SER: 21.8), while FT produced the least accurate *K*^trans^ (CCC: 0.842) and *v*_e_ (CCC: 0.799).

**Conclusion:**

TV/TGV_*α*_^2^ should be used as temporal constraints for CS DCE-MRI of the breast.

## 1. Introduction

Dynamic contrast enhanced magnetic resonance imaging (DCE-MRI) involves the continuous acquisition of *T*_1_-weighted MR images during and after the injection of a paramagnetic contrast agent (CA). The CA increases the contrast between different tissues by altering their inherent relaxation rates. Across serial images each image voxel yields an intensity time course that can be used to estimate physiological parameters, such as the volume transfer constant, *K*^trans^, and extravascular-extracellular volume fraction, *v*_e_ [1, 2]. Both high temporal and spatial resolutions are beneficial in DCE-MRI; high temporal resolution is needed for quantitative DCE-MRI analysis, while high spatial resolution aids clinical reading. However, the requirement for repeated, high signal-to-noise images limits simultaneous enhancement of temporal and spatial resolutions by conventional data acquisition methods.

For the acceleration of dynamic MR images, a common strategy used to balance the trade-off between spatial and temporal resolution is to subsample the data (also known as “*k*-space”) at each frame. Many successful algorithms have used this idea, such as keyhole [3], *k*-*t* FOCUSS (FOcal Underdetermined System Solver) [4], *k*-*t* BLAST (Broad-use Linear Acquisition Speed-up Technique), and *k*-*t* SENSE (SENSitivity Encoding) [5]. However these methods suffer the limitations such as low signal-to-noise ratio (SNR) and aliasing artifacts for high sampling factors.

Compressed sensing (CS) [6, 7] is a newer strategy to accelerate data acquisition in dynamic MRI. Using CS, it is possible to accurately reconstruct an MR image from less Fourier data than with traditional acceleration methods, resulting in a further reduced data collection burden [8, 9]. According to CS theory, the reconstruction of dynamic MR images can be modeled as a constrained reconstruction in which the resulting image is chosen to have the sparsest representation in some prior sparse transform while still being consistent with the collected Fourier data. For dynamic images, since most of the image is roughly constant in time, the most significant redundancy (and hence sparsity) often manifests in the temporal direction.

CS has great potential in cancer MRI [10] because many protocols in cancer MRI are dynamic. Many applications of the CS to dynamic MRI have been successfully demonstrated, such as *k*-*t* SPARSE [11], *k*-*t* SLR (Sparse and Low Rank) [12], and iGRASP (Golden-angle RAdial Sparse Parallel MRI) [13]. For example, Han et al. [14] demonstrated the enhancement of spatiotemporal resolution of DCE-MRI in an animal model with a CS-accelerated Cartesian fast low angle shot (FLASH) sequence. Chen et al. [15] compared different forms of temporal total variation terms in the reconstruction of undersampled DCE-MRI data acquired in breast cancer patients. Ji and Lang [16] applied a difference operator to the temporal data frames to enhance the spatial signal sparsity for CS reconstruction. Smith et al. [17, 18] showed the expected variance in quantitative parameters for spatial TV regularization across a population of randomly generated sampling schemes. Although CS has been applied to breast DCE-MRI, no one has quantitatively compared different temporal sparsity models for breast DCE-MRI across a large number of sampling patterns. Thus, the expected effects of different temporal regularizers on the error in quantitative DCE-MRI parameters are not known.

Before CS can be used clinically in such a critical area of care as cancer imaging, its effect on the reliability and accuracy must be understood. The aim of this paper partially addresses this by quantitatively evaluating five common temporal sparse regularizers for breast DCE-MRI:

1. *ℓ*_1_-norm of the Fourier transform (FT)
2. *ℓ*_*l*_-norm of the Haar wavelet transform (WT)
3. Total variation (TV),
4. Second-order total generalized variation (TGV_*α*_^2^)
5. Nuclear norm (NN)

We hypothesize that one of these regularizers will produce more accurate reconstructed images than the others and that one regularizer (not necessarily the same one) will produce the most accurate quantitative parameters.

## 2. Materials and Methods

### 2.1. Data Collection

We applied all models retrospectively to in vivo breast DCE-MRI data [19] collected under a protocol approved by the institutional review board. The data were acquired using a spoiled gradient-recalled echo (SPGRE) sequence on a Philips (Best, Netherlands) Achieva 3T scanner with TR = 4.33 ms, TE = 2.12 ms, and flip angle = 12°. The dimension of the data was 192 × 192 × 10 × 105, which consisted of 192 readout points by 192 phase encodes across 10 slices repeated over 105 dynamics. The spatial resolution was 1.33 mm by 1.33 mm by 5 mm, and the field of view was 256 mm by 256 mm by 50 mm. Further details about the protocol can be found in [19].

The data that were acquired were fully sampled with a Cartesian geometry and then retrospectively undersampled according to a range of random sampling patterns. While this was less realistic than prospective undersampling, it was necessary because acquiring hundreds of different sampling patterns prospectively would be impractical.

To narrow the focus of the paper, we looked only at the slice that passed through the center of the tumor. For this particular dataset, the center slice was the sixth slice. Also, for all reconstructions, we cropped the image posterior of the chest wall to improve sparsity. The beating heart caused aliasing artifacts in the first-phase encode direction (superior-inferior). The final cropped dimensions of our test data were 192 × 128 in-plane by 105 dynamics. This cropping procedure can be guided by the undersampled data, as even on the aliased images the chest wall can be demarcated.

**Figure 1:**
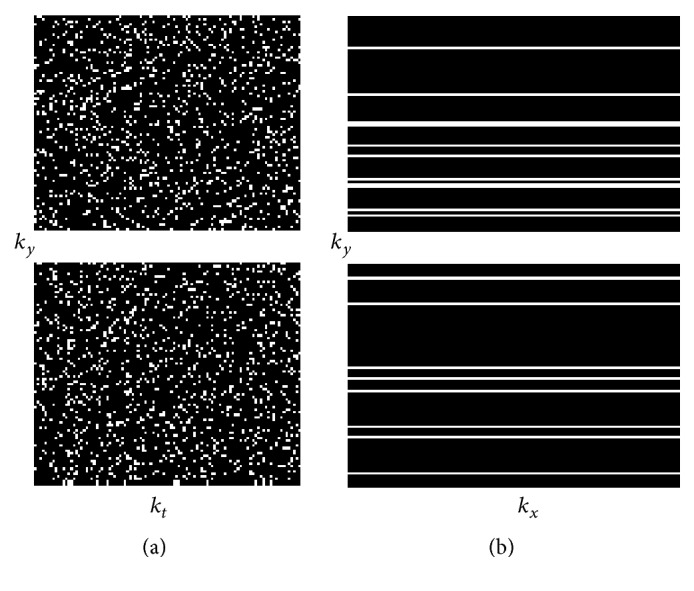
An example sampling pattern for all dynamics (a) and a 2D sampling for one dynamic (b). The 2D mask on (b) is generated by repeating one column of the mask on (a). For each dynamic, the central lines are fully sampled and the periphery is randomly sampled. The total undersampling factor is around 4.5.

We generated 200 distinct Cartesian sampling masks by choosing 200 different random seeds before pattern generation. The dimensions of the masks were 192 × 128 × 105, matching the dimensions of the data. For each dynamic, the low frequency region was fully sampled with a central window width of 20 *k*-space lines. Outside the central window, we randomly chose phase encodes such that each phase encode was sampled roughly the same number of times, but at random time points. The total undersampling factor of the mask on average was 4.5 across all 200 masks. Here we chose 4.5 because of the limitations of the Cartesian undersampling scheme. Higher undersampling factors can be achieved using non-Cartesian schemes, such as radial and spiral, which are not in the scope of this paper. The actual acceleration delivered for a given seed varied because the exact number of phase encodes chosen in a mask varied slightly due to the pseudorandom nature of the pattern generation.  Figure 1 shows an example sampling mask.

### 2.2. Image Reconstruction

We denote the reconstructed spatiotemporal image slice as the matrix *X* ∈ *ℂ*^*mn*×*d*^, where the spatial dimensions are *m* × *n* with *d* temporal dynamics. The dynamic measurements correspond to the samples in *k*-space corrupted with noise: *B* = *AX* + *ϵ*, where *A* = *Mℱ* is the measurement operator, *ℱ* is a 2D spatial Fourier transform on each temporal frame, *M* is the sampling mask on each temporal frame, and *ϵ* is additive complex white Gaussian noise.

The reconstruction problem solved was

(1) $$\begin{array}{c} \hat{X}=\underset{X}{\arg\;\min}\frac{1}{2}{\left\|\;AX-B\;\right\|}_{F}^{2}+\alpha S\left(\;X\;\right), \end{array}$$

where *B* is the collected (undersampled) data, *α* is a positive, real parameter that balances between data consistency and sparsity, *F* is Frobenius norm, and *S* is one of the five temporal sparsity-promoting regularizers. Minimizing *S*(*X*) promotes the sparsity of the outcome and minimizing ‖*AX* − *B*‖_*F*_^2^ enforces data consistency.

In this paper, all the sparse models are solved by the Fast Iterative Shrinkage-Thresholding Algorithm (FISTA) [20] for its simplicity and efficiency. FISTA is an operator splitting algorithm that aims to minimize the following problem:

(2) $$\begin{array}{c} \hat{x}=\underset{x\in C^{n}}{\arg\;\min}f\left(\;x\;\right)+g\left(\;x\;\right), \end{array}$$

where *f* is a smooth convex function with Lipschitz constant *L*_*f*_ and *g* is a convex function which may be nonsmooth. The outline of FISTA is shown in Algorithm 1.

Here prox_*τ*_(*g*)(*u*) is the proximal map of a function *g*(*x*) at point *x*, which is defined as

(3) $$\begin{array}{c} prox_{\tau }\left(\;g\;\right)\left(\;u\;\right)=\underset{x}{\arg\;\min}\;g\left(\;u\;\right)+\frac{1}{2\tau }{\left\|\;u-x\;\right\|}_{2}^{2}. \end{array}$$

And the projection operator is defined as follows:

(4) $$\begin{array}{c} x=project\left(\;x,\left[\;l,u\;\right]\;\right)=\left\{\begin{array}{cc} x, & \text{if}\;\;l\leq x\leq u;\\ l, & \text{if}\;\;x<l;\\ u, & \text{if}\;\;x>u. \end{array}\right. \end{array}$$

The key step of FISTA is to find an efficient algorithm to solve the proximal map subproblem. As in most compressed sensing models, we use *l*_1_ norm as sparsity term, and the proximal maps can be efficiently solved by soft thresholding. For example, let *g*(*u*) = ‖*u*‖_1_ in (3). Now subproblem (3) becomes

(5) $$\begin{array}{c} prox_{\tau }\left(\;g\;\right)\left(\;u\;\right)=\underset{u}{\arg\;\min}{\left\|\;u\;\right\|}_{1}+\frac{1}{2\tau }{\left\|\;u-x\;\right\|}_{2}^{2}. \end{array}$$

And the solution of (5) is given by

(6) $$\begin{array}{c} u=T_{\tau }\left(\;x\;\right), \end{array}$$

where the soft shrinkage operator *𝒯*_*τ*_ : *ℂ*^*n*^ → *ℂ*^*n*^ is defined as

(7) $$\begin{array}{c} T_{\tau }\left(\;u\;\right)=\max\left(\;0,m-\tau \;\right)\exp\left(\;i\varphi \;\right), \end{array}$$

where *m* = abs *u*, *φ* = arg⁡*u*, and “max” is the maximum operator that chooses the largest of two elements.

#### 2.2.1. Regularizer 1: *ℓ*_1_-Norm of the Fourier Transform

Dynamic MRI often shows temporal redundancy (repeated features of the signal in time), so the 1D Fourier transform applied along the temporal dimension can be used to sparsify the signal. The more periodic the signal, the sparser the representation in the Fourier domain. A nonperiodic, noisy signal can still be compressed in the Fourier domain due to the denoising effect of downsampling, although this is a minor effect. For example, in cardiac cine imaging, the image can be efficiently sparsified using a temporal Fourier transform due to the periodic motion of the heart. In *k*-*t* SPARSE [11], a wavelet on the spatial domain and a Fourier transform along the temporal direction was used to make the image sparse. Denoting *ℱ*_*t*_ as 1D temporal Fourier transform, we get the model

(8) $$\begin{array}{c} \hat{X}=\underset{X}{\arg\;\min}\frac{1}{2}{\left\|\;AX-B\;\right\|}_{F}^{2}+\alpha {\left\|\;F_{t}X\;\right\|}_{1}. \end{array}$$

#### 2.2.2. Regularizer 2: *ℓ*_*l*_-Norm of the Haar Wavelet

Wavelets are a family of sparsifying transforms commonly used in CS reconstructions. In particular for MRI, Haar wavelets have two features of interest. First, piecewise constant signals are known to be sparse under the Haar wavelet transform. And second the Haar wavelet in particular has a high mutual incoherence [8] with the Fourier transform, the domain of data collection for MRI. Based on the results of CS theory, this should lead to a better reconstruction for a given undersampling factor. For example, *k*-*t* SPARSE [11] has successfully employed spatial wavelets in cardiac imaging. Here we define *W*_*t*_ as the 1D temporal Haar wavelet transform, yielding the model

(9) $$\begin{array}{c} \hat{X}=\underset{X}{\arg\;\min}\frac{1}{2}{\left\|\;AX-B\;\right\|}_{F}^{2}+\alpha {\left\|\;W_{t}X\;\right\|}_{1}. \end{array}$$

#### 2.2.3. Regularizer 3: Total Variation

In the first application of compressed sensing in MRI, Lustig et al. [8] used total variation (TV) as the sparsifying transform in the model. The TV operator considers an equally weighted combination of finite differences along space and time, essentially representing the image in terms of its gradient. TV is maximally incoherent with the Fourier domain and as such should yield on average a more accurate reconstruction for a given undersampling factor than any other sparse basis.

However, real MR images are not piecewise constant, and constraining the gradient of these images produces “staircase” artifacts. Staircase artifacts manifest when higher-order variations in the signal intensity are reduced to piecewise constant regions. This occurs because the smallest gradient coefficients are being reduced to zero in the reconstruction in order to sparsify the image under the gradient transform.

TV or finite difference based strategies, which were originally designed for image denoising [21], have recently gained wide interest in many MRI applications beyond denoising. In *k*-*t* SLR, a three-dimensional total variation transform was used which is defined as follows:

(10) $$\begin{array}{c} {\left\|\;\cdot \;\right\|}_{TV}=\sqrt{{\left(\nabla_{x}\cdot \right)}^{2}+{\left(\nabla_{y}\cdot \right)}^{2}+{\left(\nabla_{t}\cdot \right)}^{2}}, \end{array}$$

where ∇_*x*_, ∇_*y*_, and ∇_*t*_ are finite difference operators along *x*, *y*, and *t*. Several works have successfully used temporal gradient only in CS models, such as DLTG [22] and iGRASP [13]. In this paper, we focus on the temporal gradient and evaluate the performance of the following model:

(11) $$\begin{array}{c} \hat{X}=\underset{X}{\arg\;\min}\frac{1}{2}{\left\|\;AX-B\;\right\|}_{F}^{2}+\alpha {\left\|\;\nabla_{t}X\;\right\|}_{1}. \end{array}$$

#### 2.2.4. Regularizer 4: Total Generalized Variation

As mentioned above, finite difference operators are well suited to sparsify MR images, but the first-order finite difference can introduce staircase artifacts. Total generalized variation (TGV) [23] was introduced in part to address this issue. Under the TGV, linear intensity changes in the image are preserved because the TGV operates on second-order and higher derivatives.

The discrete second-order TGV is defined as follows:

(12) $$\begin{array}{c} TGV_{\alpha }^{2}\left(\;x\;\right)=\underset{v}{\arg\;\min}\;\alpha_{1}{\left\|\;\nabla x-v\;\right\|}_{1}+\alpha_{0}{\left\|\;E\left(\;v\;\right)\;\right\|}_{1}. \end{array}$$

Here the minimum is taken over all discrete complex vector fields *v* on *V* ∈ *ℂ*^2*mn*^, ∇*v* = (∇_*x*_*v*_1_, ∇_*y*_*v*_2_) denotes the first-order finite difference, and *ℰ*(*v*) = (∇*v* + ∇*v*^*T*^)/2 denotes the symmetrized derivative. Here *v*_1_ ∈ *ℂ*^*mn*^ and *v*_2_ ∈ *ℂ*^*mn*^ are the components of *v* along the first and second direction, respectively. Such a definition provides a way of balancing the first and second derivative of the function.

As can be seen from the definition, TGV_*α*_^2^ involves higher-order derivatives to measure image features. TGV_*α*_^2^ generalizes TV and is more suitable to model intensity variations in smooth regions of the image. Reconstruction with TGV_*α*_^2^ is capable of preserving sharp edges without causing staircase artifacts.

Like the case in total variation, we denote TGV_*α*_^2^ in temporal dimension as TGV_*α*_^2^ and the model becomes

(13) $$\begin{array}{c} \hat{X}=\underset{X}{\arg\;\min}\frac{1}{2\lambda }{\left\|\;AX-B\;\right\|}_{F}^{2}+TGV_{\alpha }^{2}\left(\;X\;\right), \end{array}$$

where ∇*v* = ∇_*t*_*v* with *v* ∈ *V* ∈ *ℂ*^*mn*×*d*^.

#### 2.2.5. Regularizer 5: Nuclear Norm

Low-rank matrix completion has been applied to dynamic MRI by considering each temporal frame as a column of a spatiotemporal matrix, where the spatiotemporal correlations produce a low-rank matrix. The combination of compressed sensing and low-rank matrix completion [24] has produced further increases in imaging speed.

In dynamic MRI, previous work on this combination proposed a solution that is both low rank and sparse. In *k*-*t* SLR [12], both nuclear norm and total variation transform were used to demonstrate significant improvement in performance of phantoms and in vivo cardiac perfusion MRI data. Nuclear norm (NN) is denoted by ‖*X*‖_*∗*_, which is the sum of singular values of *X*. Here we use only temporal constraints, so the formulation is as follows:

(14) $$\begin{array}{c} \hat{X}=\underset{X}{\arg\;\min}\frac{1}{2}{\left\|\;AX-B\;\right\|}_{F}^{2}+\alpha {\left\|\;X\;\right\|}_{*}. \end{array}$$

#### 2.2.6. Parameter Selection

The choice of parameters for each sparse model was crucial, as comparisons were only possible if each model were optimized. In the experiments, the data were rescaled to [0,1] before reconstruction. This rescaling simplified the tuning process.  Table 1 shows the parameters used for all sparse models. Here *α* was the weight for *S*(*x*), “iter” was the number of iterations for the main function, *σ*, *τ*, and *λ* were the first-order step size, second-order step size, and weight of fidelity term, respectively, in TV and TGV_*α*_^2^ based models. We started with very low regularization (*α* = 10^−5^) and then increased the parameters step by step until artifacts were visually eliminated from the resulting image. In each iteration, we tracked the relative norm of the image difference between two iterations. Once it went lower than a chosen threshold (10^−3^), we terminated the reconstruction.

### 2.3. Pharmacokinetic Modeling

One of the most commonly used pharmacokinetic models is the standard Tofts-Kety model [25]. This model provides information about the influx forward volume transfer constant from plasma into the extravascular-extracellular space (EES) and fractional volume of EES per unit of tissue. The standard Tofts-Kety model, with both spatial and temporal dependencies made explicit, is

(15) CTx,t=Ktransx∫0tCPsexp⁡Ktransxs−tvexds,

where *C*_*T*_ is the concentration of CA in the tissue and *C*_*P*_ is the concentration of CA delivered by the blood plasma. In our experiments, this model was fit using nonlinear least squares [18] for every voxel in the reconstructed data that enhanced by a factor of two or more relative to the precontrast baselines, calculated by dividing the mean signal in the first three dynamics to the mean signal in the last three dynamics.

### 2.4. Assessments

We used two methods to quantitatively evaluate the temporal transforms. For consistency with previous paper [12], the first assessment we used was the image-based signal-to-error ratio (SER), defined as

(16) $$\begin{array}{c} SER=-20\;\log_{10}\frac{{\left\|\hat{X}-X_{FS}\right\|}_{F}}{{\left\|X_{FS}\right\|}_{F}}, \end{array}$$

where *X*_FS_ is the image reconstructed from the original, fully sampled data.

The second assessment was the concordance correlation coefficients (CCCs) of the parametric maps *K*^trans^ and *v*_*e*_, which is widely used in the quantitative analysis of DCE-MRI. In statistics, the CCC measures the agreement between two variables and is given by

(17) $$\begin{array}{c} CCC=\frac{2\sigma_{FS}\sigma_{CS}}{\sigma_{FS}^{2}+\sigma_{CS}^{2}+{\left(\mu_{FS}-\mu_{CS}\right)}^{2}}, \end{array}$$

where *μ*_FS_ and *μ*_CS_ are the means of *K*^trans^ or *v*_*e*_ for fully sampled and compressed sensing (CS) reconstructed images, respectively, and *σ*_FS_ and *σ*_CS_ are the corresponding variances.

To visually evaluate the accuracy in determining time profiles, we computed the difference in signal intensity curves with respect to the fully sampled data in specific regions within the tumor. For consistently plotting these curves, we first manually generated a mask for the tumor and applied the mask to all reconstructions. Additional visual assessment of parameter agreement was conducted using Bland-Altman plots of the average of the undersampled parameters and the fully sampled parameters relative to the fully sampled parameter values.

### 2.5. Implementation

The CS reconstruction was written in MATLAB, and the DCE analysis software used was DCEMRI.jl [26] and was written in Julia. All experiments were run on a dual Xeon E5-2665 2.40 GHz workstation with 20 GB of RAM with MATLAB 2015b (Mathworks, Natick, MA) and Julia 0.4.3. The full code to generate the results and figures here has been provided as open source [27].

## 3. Results and Discussions

### 3.1. Image Quality

We first evaluated the performance of the five constraints on image error. The first column of Table 2 represents the signal-to-error ratio (SER) comparisons over 200 runs. We found that NN produced the highest SER (29.1, higher is better) among the five constraints tested while WT produced the lowest (21.8). FT (26.4) came in between WT and TV. TV and TGV_*α*_^2^ produced similar SERs with TGV_*α*_^2^ (27.8) being slightly higher than TV (27.7).

**Figure 2:**
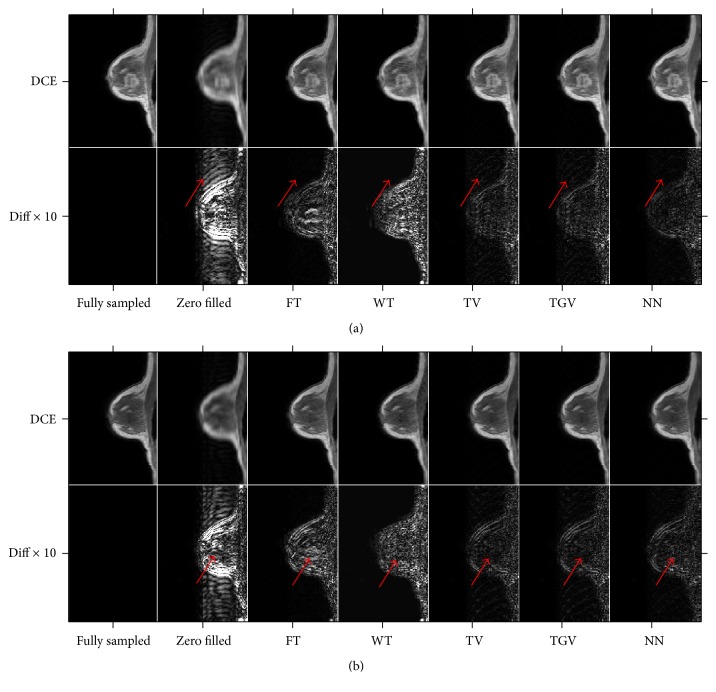
Reconstruction using the first mask for all five temporal constraints. (a) The 105th dynamic; (b) the first dynamic. In each subfigure, the first row shows the fully sampled, zero-filled, WT, FT, TV, TGV_*α*_^2^, and NN, respectively, and the second row is the corresponding difference images scaled up by a factor of 10. The aliasing artifacts are greatly reduced in the areas indicated by the arrows by TV, TGV, and NN.

Figure 2(a) shows the 105th dynamic of the reconstruction for each of the five constraints using the first randomly generated mask. The red arrows indicate background artifacts that remained in the TGV_*α*_^2^ and TV cases, where NN greatly reduced those artifacts. The background artifacts were also suppressed in the WT and FT reconstructions, but WT and FT performed the worst in reconstructing the tumor.

Figure 2(b) shows the first dynamic of the reconstructions using the five constraints for the first sampling pattern out of the 200 tested patterns. We can see from the red arrows that TV and TGV_*α*_^2^ performed visually the best in the reconstruction of tumor area, which is where the majority of voxels used in the *K*^trans^ and *v*_*e*_ calculations were located. FT performed visually the worst in the tumor area.

**Figure 3:**
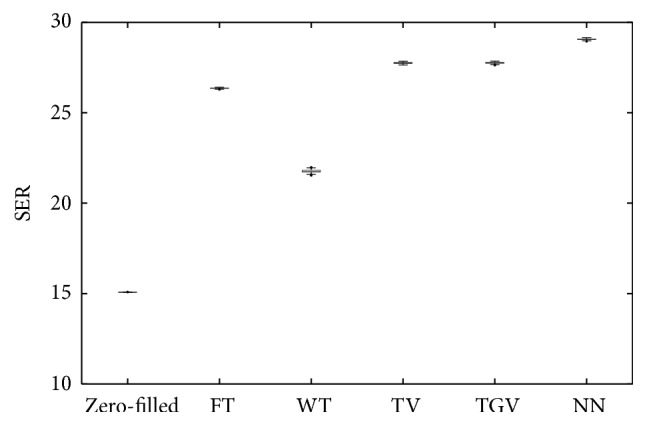
Boxplots of SER over 200 different sampling patterns. In the boxplot, the results of the five temporal regularizers are statistically different from each other except the ones between TV and TGV_*α*_^2^. We can observe that NN produced the highest SER, while WT yielded the lowest SER, which is an illustration of Table 2.

Figure 3 shows the boxplot of SER using 200 different sampling patterns for all five regularizers. We can observe from the boxplot that, for each regularizer, the variance in SER was small relative to the mean and all of the results are statistically significantly different except for TV and TGV_*α*_^2^. Also it can be seen that NN produced the highest mean SER (29.1), and WT produced the lowest mean SER (21.8).

### 3.2. Parameter Accuracy

The second and third columns of Table 2 show the CCC comparisons of *K*^trans^ and *v*_*e*_, respectively. The highest CCCs for both *K*^trans^ and *v*_*e*_ were seen using TV (0.974 for *K*^trans^ and 0.916 for *v*_*e*_) and TGV_*α*_^2^ (0.974 for *K*^trans^ and 0.917 for *v*_*e*_). Although NN produced the best SER (29.1), it did not give the highest CCCs for *K*^trans^ (0.842) and *v*_*e*_ (0.799). The same phenomenon can be found between WT and FT, where WT (21.8) produced a lower SER than FT (26.4), but WT produced a higher CCC (0.878 for *K*^trans^ and 0.733 for *v*_*e*_).

**Figure 4:**
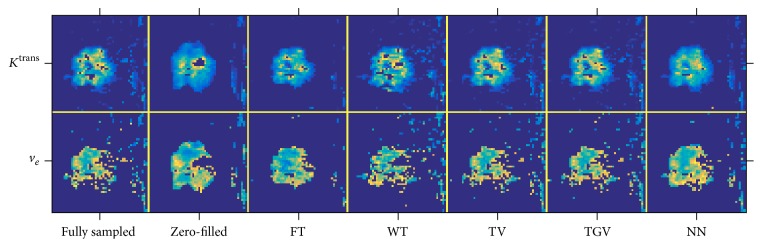
Zoomed *K*^trans^ and *v*_*e*_ maps of the first mask for all five temporal constraints. The first row shows the *K*^trans^ map for (from left to right) the fully sampled, zero-filled, WT, FT, TV, TGV_*α*_^2^, and NN. The second row is the corresponding *v*_*e*_ map. TV and TGV produced the most visually similar tumor maps to the fully sampled, without excessively denoising or filtering the image.

A zoomed image of *K*^trans^ and *v*_*e*_ maps of the first run can be seen in Figure 4. Both TV and TGV_*α*_^2^ produced accurate *K*^trans^ and *v*_*e*_ with respect to the fully sampled data, while FT produced the worst *K*^trans^ and *v*_*e*_. Although NN produced a more accurate *v*_*e*_ map than WT, the *K*^trans^ map was blurred and less accurate.

**Figure 5:**
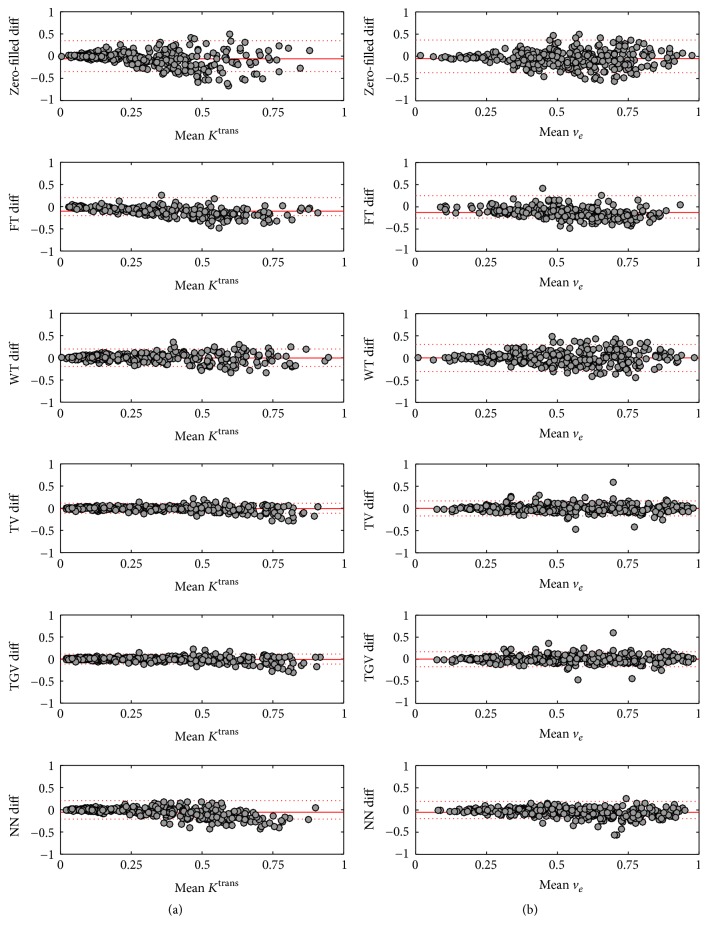
Bland-Altman plots of *K*^trans^ and *v*_*e*_ of the first mask for all five temporal regularizers. (a) is *K*^trans^, and (b) is *v*_*e*_. Rows from top to bottom correspond to WT, FT, TV, TGV_*α*_^2^, and NN, respectively. TV and TGV appear to produce the most accurate and least biased parameters. FT was the most biased, and WT was the least accurate. NN was relatively inaccurate but had only a small bias.

Bland-Altman plots of *K*^trans^ and *v*_*e*_ using the five regularizers in the first run can be seen in Figure 5. TV and TGV_*α*_^2^ produced nearly unbiased *K*^trans^ and *v*_*e*_ while WT, FT, and NN underestimated both parameters. Also although WT produced relatively accurate CCCs, it greatly underestimated the tumor means.

**Figure 6:**
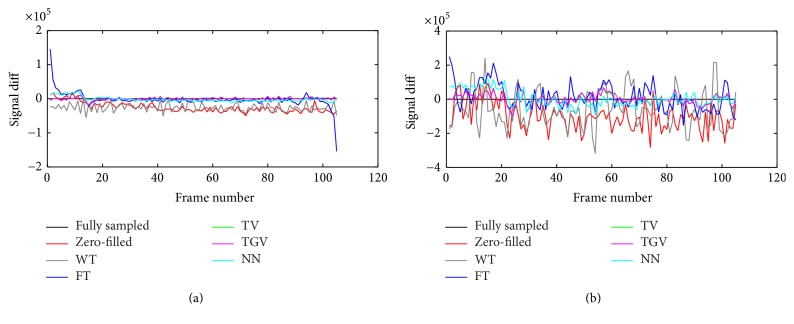
Difference plots of tumor mean intensity curves (a) and voxel intensity curves (b) in tumor area. Here the coordinate of the voxel is (96, 96). Panel (a) shows that the zero-filled reconstruction underestimated the mean intensity after CA injection. WT underestimated the mean intensity over all the dynamics. FT failed to capture the mean intensity curve in the first and last 5 dynamics. TV and TGV_*α*_^2^ best fit the mean intensity curve. NN overestimated the intensity curve before the CA injection and slightly underestimated the intensity curve after the CA injection. Panel (b) shows that zero-filled reconstruction underestimated the voxel intensity after the CA injection. FT and WT oscillated with large amplitude, showing an inaccurate approximation. Both TV and TGV_*α*_^2^ reconstructions fluctuated less. NN overestimated the signal in the preinjection period.

Figure 6 shows the difference between the mean intensity curves in the undersampled and the fully sampled cases for all voxels (panel (a)) in the tumor ROI and for a single example voxel (panel (b)). In Figure 6, the zero-filled reconstruction was accurate for the first five dynamics. But after CA injection, the intensity became underestimated, decreasing the SER, *K*^trans^, and *v*_*e*_. In Figure 6(a), WT underestimated the mean intensity curve across all the dynamics. In Figure 6(a), FT failed to fit the mean curve in the first five dynamics, and, in the last five dynamics, and the oscillation behavior is more obvious with FT in Figure 6(b). TGV_*α*_^2^ and TV best fit both the mean intensity curves and single voxel intensity curves. NN overestimated the intensity in the first few dynamics, affecting the accuracy of *K*^trans^ and *v*_*e*_.

**Figure 7:**
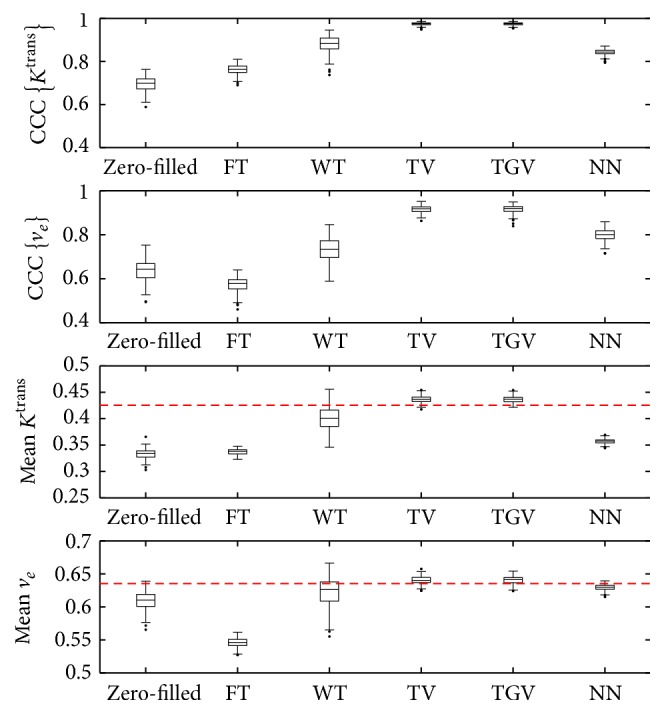
Boxplots of CCCs and tumor means over 200 different sampling patterns. In the first two boxes, the results of the five temporal regularizers are statistically different from each other except the ones between TV and TGV_*α*_^2^. We can observe TV and TGV_*α*_^2^ yielded the highest CCCs, which is an illustration of Table 2. In the last two boxes, the red horizontal lines indicate the true tumor mean *K*^trans^ and *v*_*e*_, which are 0.425 and 0.635, respectively. Consistent with the results in Table 2, TV and TGV_*α*_^2^ produced the most accurate tumor mean *K*^trans^ and *v*_*e*_ compared to the true values. Interestingly, NN tended to greatly underestimate *K*^trans^ but accurately found *v*_*e*_ on average with a slight underestimation.

Since predictable accuracy is important in breast CS DCE-MRI, standard boxplots of CCCs and the tumor mean *K*^trans^ and *v*_*e*_ are presented in Figure 7. Through all the boxplots, the interquartile ranges are small, which suggests that CS undersampling can have a predictable accuracy for Cartesian DCE-MRI of the breast. In the first three boxes (SER and CCCs), the results are all statistically significantly different except between TV and TGV_*α*_^2^. Similar to our findings in Figure 6, TV and TGV_*α*_^2^ led to the most accurate CCCs with high mean and low variance. The tumor means of both regularizers are closest to the ground truth. Again FT was the least accurate in reproducing both the tumor mean and the voxel-wise CCCs, and the tumor mean *K*^trans^ and *v*_*e*_ were far from the ground truth. Although WT produced relatively accurate tumor mean *K*^trans^ and *v*_*e*_, the variances were the highest among the five, which suggests less predictable accuracy.

### 3.3. Discussions

The quantitative comparisons of the five temporal constraints showed that NN was most capable of suppressing background artifacts and thus produced the highest SER. We believe this is due to its better artifact suppression ability and better edge preservation. The reason is that the minimization of the nuclear norm will suppress features that are not the same in all dynamics, such as interference and noise, while keeping static features, such as the breast and the tumor. Thus, at least in compressed sensing DCE-MRI of the breast, if one needs relatively higher image quality (SER in our case), nuclear norm would be a reasonable choice.

On the other hand, TV and TGV_*α*_^2^ provided the highest CCCs for *K*^trans^ and *v*_*e*_ among the five regularizers tested and also appeared to most closely match the true voxel intensity curves in the tumor area, suggesting that error in fitting the voxel intensity curves may predict the ultimate quantitative parameter accuracy. This is because TV and TGV_2_^*α*^, as opposed to the nuclear norm, better preserve high-SNR information that varies across dynamics, which in this case is the contrast enhancement in the tumor area. This produces a better reconstruction in the tumor and hence more accurate CCCs. If so, then the fit residuals may prospectively inform the parameter accuracy in cases where ground truths are not available. Thus, at least in compressed sensing DCE-MRI of the breast, if one needs to get more accurate quantitative parameters, TV and TGV_2_^*α*^ would be promising choices. Since the nuclear norm captures the background information and TV and TGV_2_^*α*^ capture the dynamic information, the combination of nuclear norm and TV/TGV_2_^*α*^ could be the best reconstruction model for breast DCE-MRI. This is also our work in the future.

The boxplots in Figure 7 show that there is no statistically significant difference between TV and TGV_*α*_^2^, so it is hard to distinguish between the utility of the two for this application. This may change for other anatomical sites; however, especially those where more regions of a roughly linear intensity gradient are present, rather than areas of mostly piecewise constant intensity, such as the breast.

The results of the quantitative comparisons presented here should inform clinical and research imaging reconstruction methods. For techniques that value image fidelity above accurate quantitative parameters, the best temporal regularizer may be the nuclear norm. For techniques that value quantitative parameter accuracy above image quality, TV or TGV should be preferred. The same data may be reconstructed multiple ways, of course.

Prior to this work, no measurements of the quantitative accuracy obtained from common temporal regularizers across a range of Cartesian sampling patterns had been made. This work addresses that gap, but with three major caveats. First, only one breast DCE-MRI data set was used: it is possible that the results will vary across subjects, although they are not expected to significantly. Second, though we obtained a fairly normal distribution of errors, the entire space of sampling patterns is astronomically large compared to the 200 tested ones here. It is improbable, but still possible, that a nonrepresentative set of sampling patterns was selected. Third, tuning the FISTA parameters to create the best reconstruction for each constraint is an inexact process. It is possible that slightly different results could be obtained with different FISTA parameters, but we have no reason to think that the general patterns would change.

## 4. Conclusion and Future Research

In this paper, we compare the quantitative performance of five temporal regularizers for CS DCE-MRI of the breast. We find that the Fourier transform is the least suitable regularizer because of the nonperiodic behavior of breast DCE-MRI data. The Haar wavelet transform was average in performance but was the least consistent in accuracy across the range of sampling patterns. The nuclear norm best suppressed background artifacts caused by undersampling, thus maximizing SER, but was less accurate in the recovery of pharmacokinetic parameters. Total variation and total generalized variation retrieved the most accurate pharmacokinetic parameters, with TGV_*α*_^2^ slightly edging out TV in both image quality and parameter accuracy.

Since the goal of CS DCE-MRI is to accurately measure tumor properties, we recommend using TV or TGV_*α*_^2^ as the temporal constraint in CS reconstructions of breast DCE-MRI.

Future work includes testing on the full 3D breast DCE-MRI datasets instead of only a single slice. Since performing the computations required for this work on the whole 3D data in MATLAB would be prohibitively slow, we intend to use state-of-the-art GPU acceleration techniques to reduce the computation time. We will also examine prospective non-Cartesian sampling schemes such as radial and spiral to explore the effect of higher acceleration on the quality of reconstructions achieved with the temporal regularizers examined here.

## Figures and Tables

**Algorithm 1:**
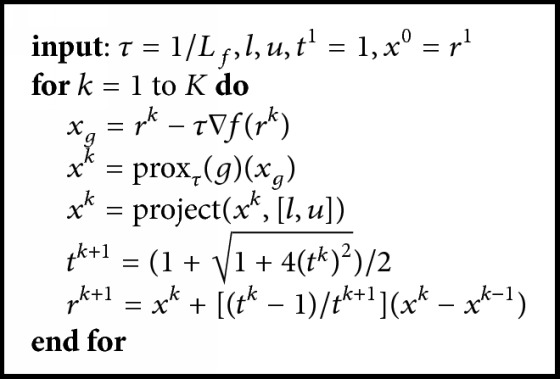
Outline of FISTA (Fast Iterative Shrinkage-Thresholding Algorithm).

**Table 1:** FISTA parameters.

Regularizer	*α*	Iter	*σ*	*τ*	*λ*
FT	0.059	35	—	—	—
WT	0.008	60	—	—	—
TV	0.5	100	0.2	0.2	0.5
TGV_*α*_^2^	0.5	100	0.2	0.2	0.5
NN	0.3	40	—	—	—

**Table 2:** Mean SER and CCC across all sampling patterns.

Constraints	SER (dB)	CCC {*K*^trans^}	CCC {*v*_*e*_}
Zero-filled	15.1	0.694	0.636
FT	26.4	0.763	0.575
WT	21.8	0.878	0.733
TV	27.7	0.974	0.916
TGV	27.8	0.974	0.917
NN	29.1	0.842	0.799

## References

[1] Choyke P. L., Dwyer A. J., Knopp M. V. (2003). Functional tumor imaging with dynamic contrast-enhanced magnetic resonance imaging. *Journal of Magnetic Resonance Imaging*.

[2] Yankeelov T. E., Luci J. J., Lepage M. (2005). Quantitative pharmacokinetic analysis of DCE-MRI data without an arterial input function: a reference region model. *Magnetic Resonance Imaging*.

[3] Van Vaals J. J., Brummer M. E., Thomas Dixon W. (1993). “Keyhole” method for accelerating imaging of contrast agent uptake. *Journal of Magnetic Resonance Imaging*.

[4] Jung H., Sung K., Nayak K. S., Kim E. Y., Ye J. C. (2009). K-t FOCUSS: a general compressed sensing framework for high resolution dynamic MRI. *Magnetic Resonance in Medicine*.

[5] Tsao J., Boesiger P., Pruessmann K. P. (2003). k-t BLAST and k-t SENSE: Dynamic MRI With High Frame Rate Exploiting Spatiotemporal Correlations. *Magnetic Resonance in Medicine*.

[6] Donoho D. L. (2006). Compressed sensing. *Institute of Electrical and Electronics Engineers. Transactions on Information Theory*.

[7] Cand\`es E. J., Romberg J., Tao T. (2006). Robust uncertainty principles: exact signal reconstruction from highly incomplete frequency information. *Institute of Electrical and Electronics Engineers. Transactions on Information Theory*.

[8] Lustig M., Donoho D., Pauly J. M. (2007). Sparse MRI: the application of compressed sensing for rapid MR imaging. *Magnetic Resonance in Medicine*.

[9] Lustig M., Donoho D. L., Santos J. M., Pauly J. M. (2008). Compressed sensing MRI: A look at how CS can improve on current imaging techniques. *IEEE Signal Processing Magazine*.

[10] Smith D. S., Li X., Abramson R. G., Quarles C. C., Yankeelov T. E., Welch E. B. (2013). Potential of compressed sensing in quantitative MR imaging of cancer. *Cancer Imaging*.

[11] Lustig M., Juan M. S., Donoho D., Pauly J. M. kt SPARSE: high frame rate dynamic MRI exploiting spatio-temporal sparsity.

[12] Lingala S. G., Hu Y., Dibella E., Jacob M. (2011). Accelerated dynamic MRI exploiting sparsity and low-rank structure: k-t SLR. *IEEE Transactions on Medical Imaging*.

[13] Feng L., Grimm R., Block K. T. O. (2014). Golden-angle radial sparse parallel MRI: combination of compressed sensing, parallel imaging, and golden-angle radial sampling for fast and flexible dynamic volumetric MRI. *Magnetic resonance in medicine*.

[14] Han S., Paulsen J. L., Zhu G. (2012). Temporal/spatial resolution improvement of in vivo DCE-MRI with compressed sensing-optimized FLASH. *Magnetic Resonance Imaging*.

[15] Chen L., Schabel M. C., DiBella E. V. R. (2010). Reconstruction of dynamic contrast enhanced magnetic resonance imaging of the breast with temporal constraints. *Magnetic Resonance Imaging*.

[16] Ji J., Lang T. Dynamic MRI with compressed sensing imaging using temporal correlations.

[17] Smith D. S., Welch E. B., Li X. (2011). Quantitative effects of using compressed sensing in dynamic contrast enhanced MRI. *Physics in Medicine and Biology*.

[18] Smith D. S., Li X., Gambrell J. V. (2012). Robustness of quantitative compressive sensing MRI: The effect of random undersampling patterns on derived parameters for DCE- and DSC-MRI. *IEEE Transactions on Medical Imaging*.

[19] Li X., Abramson R. G., Arlinghaus L. R. (2015). Multiparametric magnetic resonance imaging for predicting pathological response after the first cycle of neoadjuvant chemotherapy in breast cancer. *Investigative Radiology*.

[20] Beck A., Teboulle M. (2009). A fast iterative shrinkage-thresholding algorithm for linear inverse problems. *SIAM Journal on Imaging Sciences*.

[21] Rudin L. I., Osher S., Fatemi E. (1992). Nonlinear total variation based noise removal algorithms. *Physica D. Nonlinear Phenomena*.

[22] Caballero J., Price A. N., Rueckert D., Hajnal J. V. (2014). Dictionary learning and time sparsity for dynamic MR data reconstruction. *IEEE Transactions on Medical Imaging*.

[23] Knoll F., Bredies K., Pock T., Stollberger R. (2011). Second order total generalized variation (TGV) for MRI. *Magnetic Resonance in Medicine*.

[24] Otazo R., Candès E. J., Sodickson D. K. (2015). Low-rank plus sparse matrix decomposition for accelerated dynamic MRI with separation of background and dynamic components. *Magnetic Resonance in Medicine*.

[25] Tofts P. S., Brix G., Buckley D. L. (1999). Estimating kinetic parameters from dynamic contrast-enhanced T1-weighted MRI of a diffusable tracer: standardized quantities and symbols. *Journal of Magnetic Resonance Imaging*.

[26] Smith D. S., Li X., Arlinghaus L. R., Yankeelov T. E., BrianWelch E. (2015). DCEMRI.jl: A fast, validated, open source toolkit for dynamic contrast enhanced MRI analysis. *PeerJ*.

[27] Wang D., Arlinghaus L. R., Yankeelov T. E., Yang X., Smith D. S. https://github.com/chixindebaoyu/qetsr.

